# Initial and Middle-Term Outcome of Treatment for Spontaneous Isolated Dissection of Superior Mesenteric Artery

**DOI:** 10.1097/MD.0000000000002058

**Published:** 2015-11-13

**Authors:** Zilun Li, Huanyu Ding, Zhen Shan, Jianliang Du, Chen Yao, Guangqi Chang, Shenming Wang

**Affiliations:** From the Division of Vascular Surgery, The First Affiliated Hospital of Sun Yat-sen University (ZL, ZS, CY, GC, SW); Department of Cardiology, Guangdong Cardiovascular Institute, Guangdong General Hospital, Guangdong Academy of Sciences (HD); and Department of Medical Record Management, The First Affiliated Hospital of Sun Yat-sen University, Guangzhou, China (JD).

## Abstract

Symptomatic isolated dissection of the superior mesenteric artery (SIDSMA) represents an extremely rare condition. Although various treatments including conservative treatment, endovascular stenting (ES), and surgical repair are currently available, consensus treatment guideline is absent due to scarce of SIDSMA cases. Thus, we present our experience in the treatment of SIDSMA at our single center.

Fourteen cases of SIDSMA were treated with conservative treatment, catheter-directed thrombolysis (CDT), endovascular stenting (ES), or surgical repair at our center between January 2008 and January 2014. Demographics, clinical manifestations, coexisting medical conditions, imaging feature, treatments, and follow-up outcome of these patients were retrospectively collected and analyzed.

For 13 patients without peritonitis, conservative treatment was given for 4 to 6 days initially. After the first observation cycle, symptoms and signs were alleviated in 8 patients, and conservative treatments were continued. The remaining 5 patients received technically and clinically successful ES (in 4) or CDT (in 1) due to worsening symptoms and signs during conservative treatment. One patient with peritonitis underwent emergency surgery, with the necrotic small intestine resected. However, the abdominal pain was not alleviated 17 days postoperatively, ES was thus performed and symptoms relieved immediately. Two weeks after ES, a new aneurysm and partial thrombosis in the distal part of the stent were found by computed tomography angiography in this patient. No intestinal infarction or mortality developed during hospitalization. Follow-up was accomplished in 11 cases, ranging from 4 to 74 months (23.5 ± 21.3). Except that one complained with mild abdominal pain, the other 10 achieved complete remission. All patients were free from new aneurysmal formation of SMA and all stents remained patent.

For SIDSMA without peritonitis, conservative treatment can be provided with reasonable success rate, while ES may serve as an effective alternative once conservative treatment fails. For SIDSMA with peritonitis, open surgery remains the treatment of choice by resection of necrotic intestine and revasculization.

## INTRODUCTION

Symptomatic spontaneous isolated dissection of the superior mesenteric artery (SIDSMA) is defined as a superior mesenteric artery (SMA) dissection without the presence of the aortic dissection.^1^ SIDSMA used to be regarded as an extremely rare condition. Currently, there have been an increasing number of reports, due to the widespread use of computed tomography (CT) imaging for abdominal pain.^2^ Various treatment options, including conservative treatment, endovascular stenting (ES), and open surgery, are currently available, aiming to relieve the clinical symptoms and, more importantly, to prevent the potential intestinal infarction. However, there is no consensus on the optimal treatment strategy for SIDSMA due to rarity of cases.^1,3^ In this study, we present the short- to mid-term therapeutic outcomes of SIDSMA in our center.

## METHODS

### Data Collection

Medical records of SIDSMA patients treated in our center between January 2008 and January 2014 were collected. The information collected, including age, sex, medical history, clinical manifestations, coexisting medical conditions, imaging feature, treatment modalities, and follow-up records, was retrospectively analyzed. Because the present study was conducted retrospectively and it would not violate patients’ rights and interests in the whole process, ethical approval was not necessary and informed consent was not given.

### Diagnosis

Computed tomography angiography (CTA) was performed for all patients. SIDSMA was diagnosed according to the clinical manifestation and the abdominal CTA findings with the presence of isolated SMA wall dissection. The entry and re-entry sites of the dissection, dissection length, patency, and degree of luminal stenosis at the dissected segment of the SMA were assessed on CTA images. We categorized SIDSMA into 4 types according to Yue's classification^4^ Type I: patent true and false lumen that show entry and re-entry sites; type II: patent true lumen but no re-entry flow from the false lumen; type IIa: visible false lumen but no visible re-entry site (blind pouch of false lumen); type IIb: no visible false luminal flow (thrombosed false lumen), which usually causes true luminal narrowing; and type III: SMA dissection with occlusion of SMA.

### Treatment Options

**FIGURE 1:**
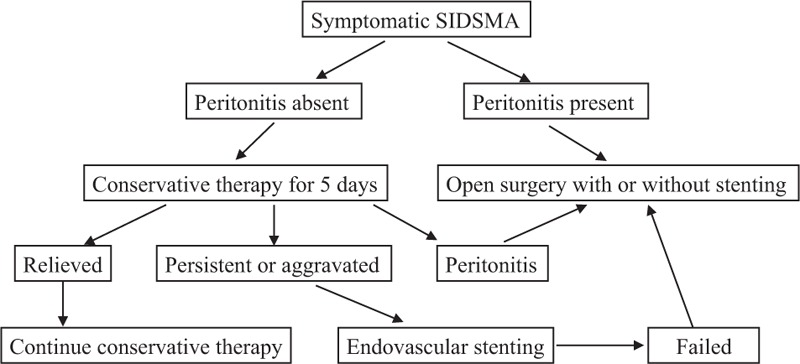
Treatment algorithm of symptomatic SIDSMA.

Conservative, endovascular, or surgical treatment was selected based on the symptoms, signs, and the morphologic characteristics of SIDSMA on CTA. For the patients with peritonitis, emergency surgery would be performed to resect necrotic bowels and restore the blood supply. For the patients without peritonitis, conservative therapy including fasting, parenteral nutrition, and antiplatelet would be given for 4 to 6 days. If symptoms were relived, conservative therapy would be continued. If symptoms persisted, endovascular stenting or open surgery would be performed. Patients undergoing antiplatelet treatment were provided with plavix 75 mg per day and aspirin 100 mg per day, while rivaroxaban 20 mg per day or low molecular heparin 4000 U twice per day was used for anticoagulant treatment. Our treatment algorithm is shown in Figure 1.

### Follow-Up

Outpatient clinic visits or telephone follow-ups were performed at 1, 6 months and annually thereafter at which time CTA findings and clinical manifestations were obtained to assess the outcome of treatment.

### Statistical Analysis

The continuous variables were expressed as mean and range. Categorical variables were recorded as number and the percentage of patients.

## RESULTS

Fourteen patients were diagnosed with SIDSMA during the study period in our center. Duration from onset to admission ranged from 0.5 day to 60 days, with a mean length of 13.8 days. All patients had abdominal pain and 2 of them accompanied with bloody stool. Tenderness found in 6 patients was the most common sign. In addition, peritonitis was detected in 1 patient. Hypertension was confirmed in 4 patients and was the most common coexisting medical condition. Use of tobacco was found in 2 patients. No patients were diagnosed with diabetes, blood lipid disorders, chronic kidney disease, coronary artery disease, or peripheral artery disease.

As measured on CTA image, the dissection had a mean length of 50.6 mm (range, 24–80 mm) and the mean distance from the orifice of SMA was 22 mm (range, 10–40 mm). According to Yue's classification, 4 patients were Type IIa SIDSMA, 9 patients were Type IIb SIDSMA, and 1 patient was Type III SIDSMA. The overview of 14 patients with SIDSMA is summarized in Table 1.

**TABLE 1:**
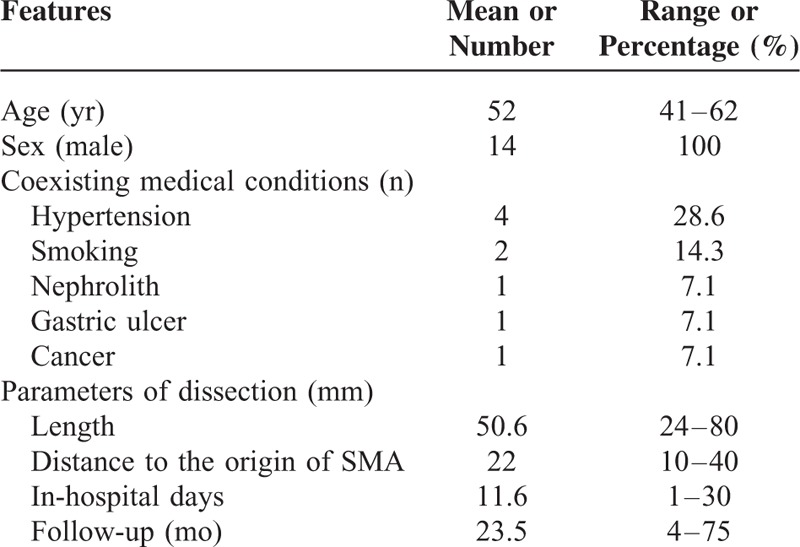
Overview of 14 Patients With SIDSMA

**FIGURE 2:**
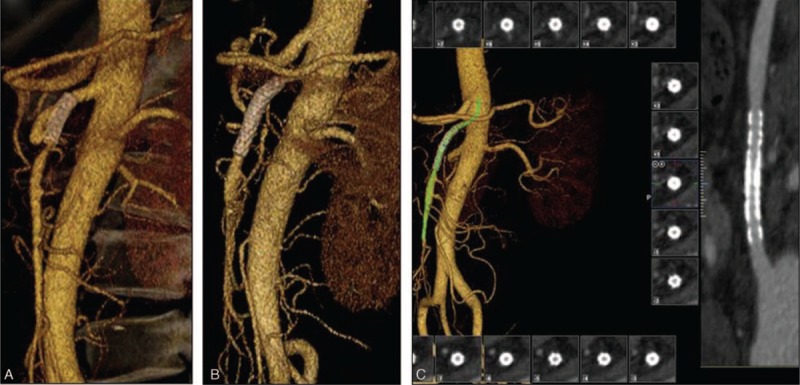
A, Two weeks after endovascular stenting, the proximal stent was patent while the distal part formed a new aneurysm and thrombosis. B, Six months later, the aneurysm and the thrombosis disappeared and the SMA was patent. C, Twenty-one months later, the SMA was patent without aneurysm or thrombosis.

Among 14 SIDSMA cases, 1 patient (no. 6) who had peritonitis underwent the emergency exploratory laparotomy. Enterectomy was performed because of the terminal ileum necrosis. Seventeen days after the surgery, abdominal pain was not alleviated in this patient. Since intestinal ischemia was suspected, DSA was performed. Stenosis of SMA was confirmed and a stent was implanted across the stenosis during the procedure. After stent implantation, the blood flow in SMA was restored and the symptoms relieved. Two weeks after endovascular treatment, the proximal part of the stent was patent while the distal part formed a new aneurysm and thrombosis according to the CTA (Figure 2A). We did not take any further intervention because the patient was free from abdominal pain or any other symptoms and signs. CTA at 6 months showed that both the aneurysm and the thrombosis disappeared and the SMA was patent (Figure 2B). During the follow-up, there were no symptoms and signs. The SMA was patent at 21 months postoperatively (Figure 2C).

For the other 13 patients without symptoms or signs of intestinal ischemia, necrosis, or aneurysms rupture, conservative treatment was given for an observation cycle of 4 to 6 days. After the first observation cycle, symptoms and signs were alleviated or at least did not worsen in 8 patients and conservative treatment was continued (Table 2). And significant relief of symptoms and signs was achieved in these 8 patients upon discharge from hospital and during a follow-up of 4 to 75 months (average of 32.7). For the other 5 patients whose symptoms and signs were aggravated, endovascular stenting with or without catheter directed thrombolysis was performed. The stents’ number, brand, size are summarized in Table 3. The technical success rate was 100%, with blood flow restored in SMA in all 5 patients. No access site complications occurred. The symptoms and signs relieved immediately after the procedure. The stent was patent in all the patients until the end of follow-up.

**TABLE 2:**
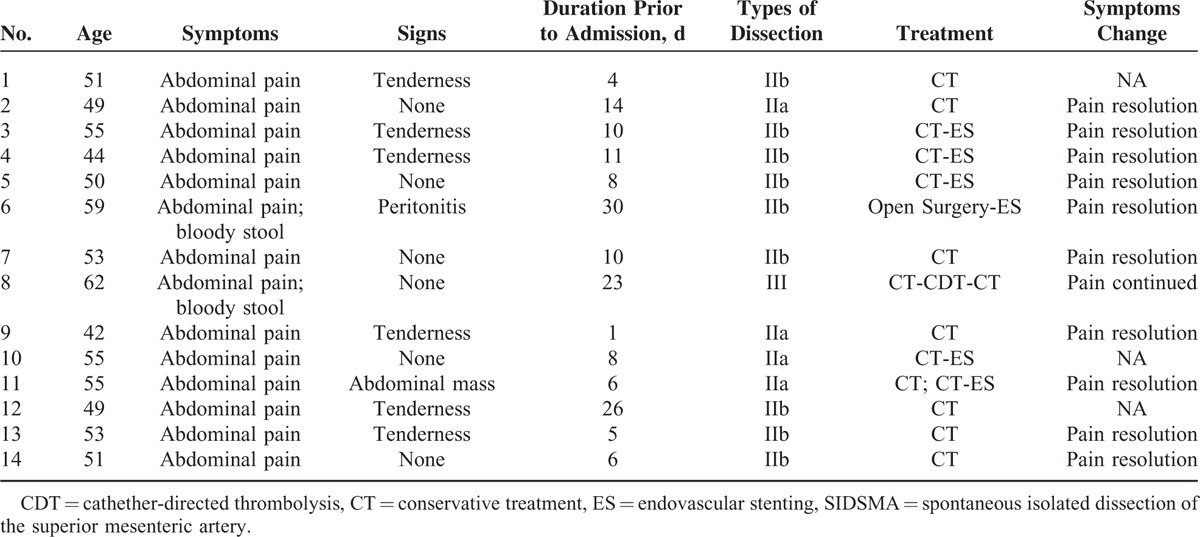
Individual Characteristics and Treatments of 14 Patients With SIDSMA

**TABLE 3:**
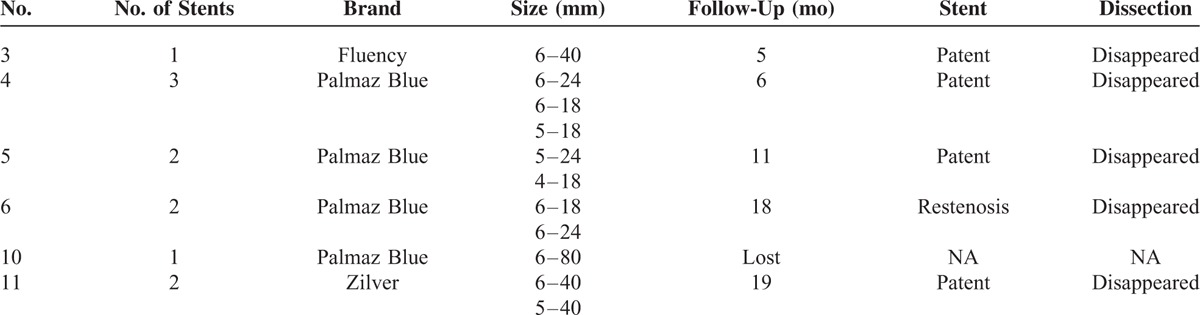
Detailed Information of Stented Patients

## DISCUSSION

The first case of SIDSMA was reported by Bauersfeld in 1947.^5^ In a series of 6666 autopsy cases, the incidence of SIDSMA was 0.06%.^6^ However, the incidence of SIDSMA might be underestimated.^7^ Luan et al reviewed literatures regarding SIDSMA published since 1947 and found SIDSMA had been reported more frequently in recent years.^7^ It might be due to the increasing use of advanced imaging technology examination such as ultrasounds or contrast-enhanced CT scans for abdominal patients.

According to Luan's review, men accounted for 84.2% of the SIDSMA cases reported since 1947 with a mean age of 55.4.^7^ Yun, Jia, and Dong also reported that SIDSMA occurred mainly in male patients in their fifth decade.^1,3,4^ In our study, all of the 14 patients were men with a mean age of 52. However, why men are predisposed to SIDSMA in their fifties remains unclear.

The underlying cause of SISMAD has not been identified because the pathologic assessment was not obtained in most patients. Luan et al^7^ reviewed 278 patients with SIDSMA reported in English language and found that the potential risk factor included hypertension, smoking, hyperlipidemia, coronary heart disease, atherosclerosis, diabetes mellitus, and trauma. To explore the mechanism of SIDSMA, Solis et al^8^ focused on the location of entry site of dissection. It was usually located at 1.5 to 3 cm distal to the orifice of SMA. Park et al^9^ found unusual mechanical stresses on the anterior wall of this portion through a computer-simulation model, and it may be an important factor in the development of SIDSMA.

Lack of specific clinical manifestations, the misdiagnosis rate of SIDSMA was relatively high. In addition, asymptomatic cases even accounted for approximately one-fourth of SIDSMA patients,^9^ rendering the diagnosis more difficult. These asymptomatic SIDSMA patients were coincidentally diagnosed by CT which was performed for other reasons. To makes the diagnosis in time, we proposed that SIDSMA should be suspected in patients whose severe abdominal symptoms were inconsistent with their mild signs. Patients with abdominal symptoms that were obscure or aggravated after conservation therapy should receive CTA or other invasive examinations in order to clarify the etiology and facilitate further interventions.

The major complications of SIDSMA were arterial rupture with bleeding and bowel necrosis because of mesenteric ischemia.^2,10–12^ Thus, the aim of treatment for SIDSMA is to limit the extension of dissection, to prevent the rupture of the false lumen, and to preserve the distal blood perfusion through the true lumen.^1^

In terms of conservative treatment for SIDSMA, there is no consensus on the duration of antiplatelet and/or anticoagulation therapy. Cho et al^13^ advised that it was necessary to be continued until the dissection disappeared. However, Yun et al^4^ insisted that there was no significant difference between the 2 groups with or without anticoagulation therapy in their study including 28 cases with SIDSMA. In out center, we gave both anticoagulation and antiplatelet therapy during hospitalization and only antiplatelet after discharge for 3 to 6 months in order to prevent thrombosis in the true lumen. For these patients, there was no thrombosis in SMA as confirmed by CTA during follow-up. Nevertheless, we could not conclude that the SIDSMA patients benefit from antiplatelet and anticoagulation therapy, due to small sample and lack of placebo control group.

Endovascular stent placement in the management of SIDSMA was first described by Leung et al.^14^ Although it is becoming more and more popular due to the efficacy, feasibility, and mini-invasion, its long-term results have yet to be determined. It seems that symptoms and signs may relieve sooner in patients treated by initial stent placement than those who received conservation treatment according to our observation. However, stent thrombosis and/or restenosis should raise special attention.

Then, what were the indications of endovascular treatment for symptomatic SIDSMA patients? Dong et al^1^ advised that endovascular treatment should be performed for patients whose abdominal symptoms did not relieve after conservation treatment. However, the role of lesion characteristic in treatment option seemed to be underestimated in their study, contributing to high rate of conversion to open surgery. During the same period, Jia et al^3^ proposed that endovascular treatment should be conserved for patients who had compression of the true lumen or dissecting aneurysm likely to rupture. In their study, morphological characteristic seemed to be overemphasized, and only Type IIA patients underwent endovascular treatment.

In our series, both lesion characteristic and abdominal symptoms were taken into consideration. Morphological criteria became more liberal and endovascular treatment was considered for Type IIB patients as well. Based on our results, we suggested that all Type II patients, who carried a high risk of arterial rupture and bowel necrosis or who did not benefit from conservation treatment, should be provided with endovascular stent placement. As for Type III patients, catheter-directed thrombolysis should be preferentially considered.

Surgical treatment in the management of SIDSMA was first reported by Sisteron and Vieville.^15^ Due to the invasion and higher incident of postoperative complications, surgical treatment was generally reserved for patients with suspected intestinal necrosis. In our study, only 1 patient received operation but he was changed to endovascular stenting 17 days after surgical treatment.

Evidence from our study further confirmed that comprehensive therapy including conservation treatment, endovascular treatment, and surgical treatment was safe and effective for SIDSMA patients. Nevertheless, there are several limitations to our study that deserve mention. The number of patients was small and the duration of follow-up was short. Furthermore, 21.4% patients were lost to follow-up, impairing the reliability of the outcomes.

## CONCULSIONS

For SIDSMA without peritonitis, conservative therapy can be provided with reasonable success rate, while ES may serve as an effective alternative once conservative therapy fails. For SIDSMA with peritonitis, open surgery remains the treatment of choice by resection of necrotic intestine and revasculization.
